# Early‐term clinical and radiographic outcomes of a new short cementless stem for primary total hip arthroplasty in young and active patients

**DOI:** 10.1002/jeo2.70729

**Published:** 2026-05-18

**Authors:** Marco Minelli, Vincenzo Longobardi, Marco Rosolani, Alessio D'Addona, Vincenzo Paolo Di Francia, Mattia Andreotti, Giangregorio Madonna, Federico Della Rocca

**Affiliations:** ^1^ Department of Biomedical Sciences Humanitas University Pieve Emanuele, Milan Italy; ^2^ IRCCS (Istituto di Ricovero e Cura a Carattere Scientifico) Humanitas Research Hospital Rozzano, Milan Italy

**Keywords:** cementless fixation, femoral prosthesis, hip arthroplasty, short femoral stem, young adult

## Abstract

**Purpose:**

To evaluate implant survivorship, complication rates and clinical and radiographic outcomes of a novel cementless short femoral stem used in primary total hip arthroplasty (THA).

**Methods:**

This monocentric retrospective study included 200 consecutive patients younger than 65 years undergoing primary unilateral THA with a cementless short femoral stem and a minimum follow‐up of 2 years. All procedures were performed by a single fellowship‐trained surgeon using a posterolateral approach. Clinical outcomes were assessed using the modified Harris Hip Score (mHHS), Western Ontario and McMaster Universities Osteoarthritis Index (WOMAC) and Visual Analogue Scale (VAS). Radiographic analysis included stem alignment, subsidence, fixation, stress shielding, radiolucent lines (RLLs), cortical hypertrophy and heterotopic ossification. Implant survivorship was estimated using Kaplan–Meier analysis, with revision for any cause as the endpoint.

**Results:**

At a mean follow‐up of 36.2 months (range: 24.0–44.0), stem survivorship was 100.0%, with no femoral components revised. One patient (0.5%) underwent head exchange for instability. Complication and reoperation rates were 1.5% and 1.0%, respectively. No femoral fractures, subsidence or aseptic loosening were observed. All clinical scores improved significantly: mHHS increased from 67.6 to 97.8, WOMAC from 60.5 to 1.5 and VAS from 7.6 to 0.3 (*p* < 0.0001). Return to sport was achieved in 85.0% of patients. Radiographically, 11.0% of stems were in varus and 5.0% in valgus. RLLs <2 mm occurred in 23.0% of cases without loosening. Stress shielding was limited (Grade I–II in 15.0%), cortical hypertrophy occurred in 4.0% and spot welds in 32.0% of patients.

**Conclusion:**

This short femoral stem demonstrated excellent early survivorship with reliable metaphyseal fixation and low complication rates, supporting its use as a safe bone‐preserving option in young and active patients.

**Level of Evidence:**

Level IV.

**Abbreviations**
BMI: body mass index
CCD: Caput–Collum–Diaphyseal
ICC: intraclass correlation coefficient
IRB: Institutional Review Board
mHHS: modified Harris Hip Score
RLLs: radiolucent lines
THA: total hip arthroplasty
Ti6Al4V: titanium–aluminium–vanadium alloy
TiCaP: titanium–calcium phosphate
VAS: Visual Analogue Scale
WOMAC: Western Ontario and McMaster Universities Osteoarthritis Index

## INTRODUCTION

Total hip arthroplasty (THA) increasingly serves younger and active patients, amplifying the importance of femoral reconstruction that preserves bone stock and maintains proximal loading [3]. Short cementless stems were conceived to achieve metaphyseal fixation, promote proximal force transfer and reduce stress shielding while simplifying future revision [38]. Classifications group short stems by their fixation site and load transfer (neck‐preserving, calcar‐loading, metaphyseal fit‐and‐fill and shortened tapered designs), underlining the heterogeneity of this implant family [18]. Neck‐preserving implants achieve fixation at the femoral neck; calcar‐loading stems are engineered to rest on the medial calcar and promote proximal load transfer; metaphyseal fit‐and‐fill stems engage the metaphysis to provide reliable stability and shortened tapered designs are abbreviated versions of conventional tapered stems, combining metaphyseal fixation with limited diaphyseal contact [25]. Since short stems accommodate the individual morphology of the proximal femur, they are more sensitive to malalignment, which could predispose to subsidence and early loosening [21, 23, 35]. Moreover, short stems require proximal loading and tight metaphyseal engagement: in osteoporotic bone, this can lead to intraoperative fractures, and without adequate cortical contact, short stems can settle more than conventional stems, potentially leading to early subsidence [33, 35]. Early‐ to mid‐term studies on short‐stem THA have consistently demonstrated excellent clinical outcomes, high patient satisfaction and survivorship rates comparable to those of conventional stems, supporting their use in younger and active patients [15, 22, 34]. However, concerns still persist regarding early mechanical complications, as well as the true extent to which short stems prevent proximal stress shielding [4, 32, 37]. There remains a need for implant‐specific, real‐world series with standardized clinical and radiographic endpoints performed under consistent technique. In this scenario, the primary objective of this study was to report the survivorship, complication and revision rates of a new short cementless hip stem in primary THA at minimum 2‐year follow‐up. The secondary aim was to record clinical and radiographic outcomes.

## MATERIALS AND METHODS

This is a monocentric retrospective study on a consecutive series of patients undergoing THA. All the surgeries were performed by a single high‐volume adult reconstruction fellowship‐trained orthopaedic surgeon. Ethical approval was obtained from the independent Institutional Review Board of IRCCS Humanitas Research Hospital (protocol no. 618/17), and written informed consent was collected from all participants. Patients were eligible for inclusion if they were younger than 65 years at the time of surgery and had undergone primary unilateral THA with cementless fixation using a short femoral stem, with complete clinical and radiographic documentation and a minimum follow‐up of 2 years. Patients were excluded if they were 65 years of age or older, had undergone revision or bilateral procedures, received cemented fixation or standard‐length or conical femoral stems, had incomplete clinical or radiographic records, had insufficient follow‐up or were unwilling or unable to provide informed consent or participate in follow‐up evaluations. A total of 200 patients (131 men and 69 women) fulfilled the inclusion criteria and underwent primary unilateral THA with a cementless short stem from April 2021 to October 2023. The mean age at surgery was 50.7 ± 8.7 years (range: 17.0–65.0). Mean body mass index was 26.4 ± 4.0 (range: 16.2–33.8). Indications for THA included primary osteoarthritis (189 cases, 93.5%), avascular necrosis (9 cases, 4.5%) and post‐traumatic osteoarthritis (2 cases, 1.0%). Patients characteristics are detailed in Table 1.

**Table 1:** Baseline demographic data and surgical indications of the study population.

Characteristics	*N* (%)
Patients	200 (100.0%)
Males	131 (65.5%)
Females	69 (34.5%)
Mean age, years (range)	50.7 ± 8.7 (17.0–65.0)
Mean BMI kg/m^2^ (range)	26.4 ± 4.0 (16.2–33.8)
Indication for THA	
Primary osteoarthritis	189 (93.5%)
Avascular necrosis	9 (4.5%)
Post‐traumatic osteoarthritis	2 (1.0%)

Abbreviations: BMI, body mass index; THA, total hip arthroplasty.

### Surgical procedure

**Figure 1:**
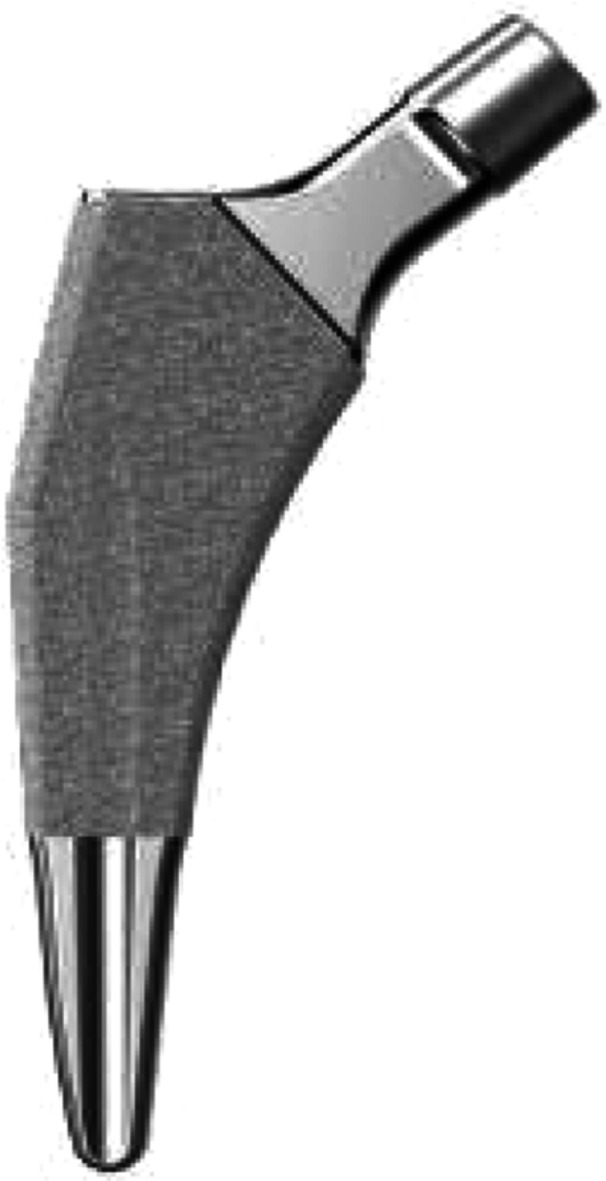
3C Hip Prosthesis System cementless short stem. Reproduced with permission from Link Italia S.p.A.

**Figure 2:**
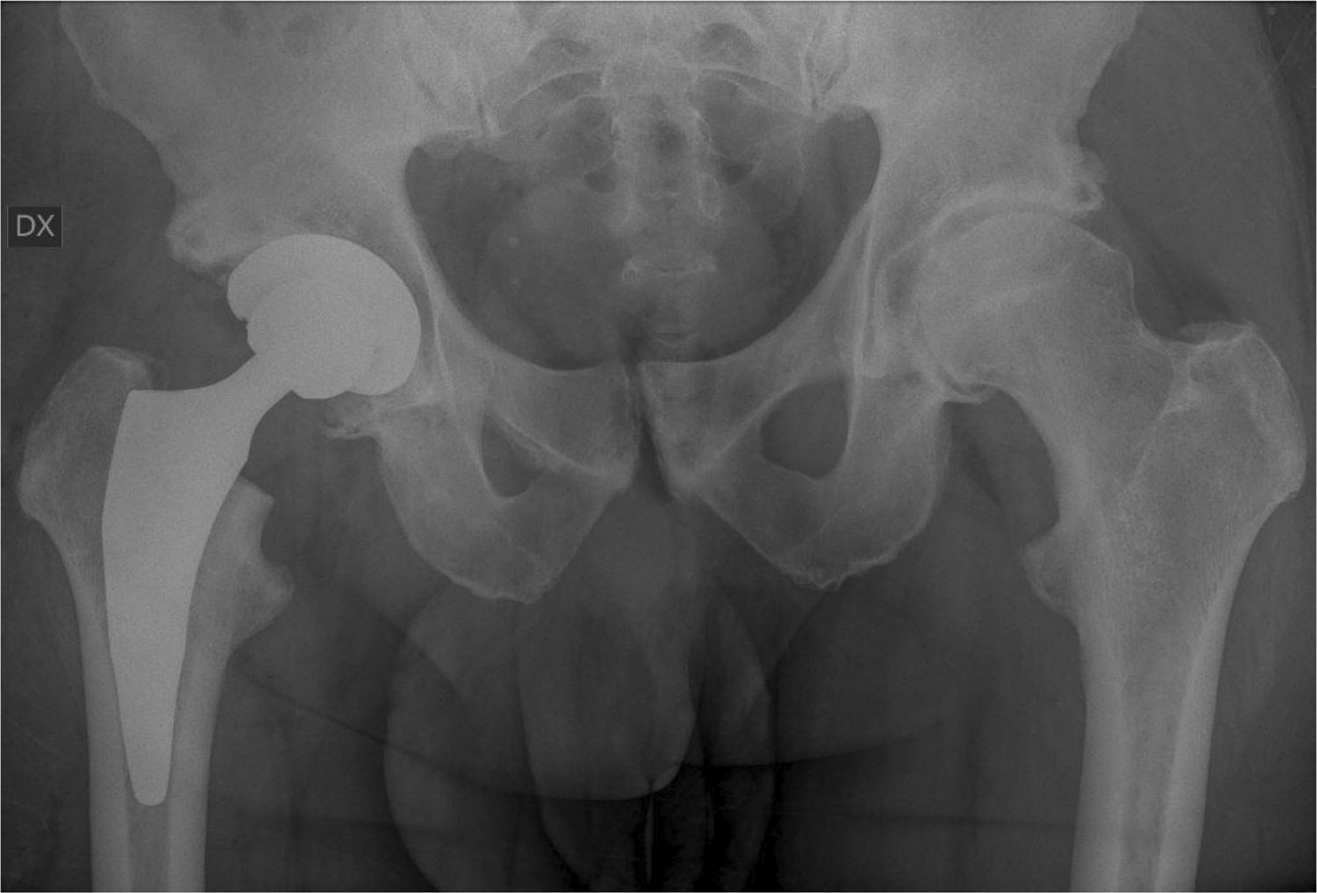
Two‐year postoperative radiographic follow‐up of a standard 3C cementless short femoral stem (CCD angle: 131°). CCD, Caput–Collum–Diaphyseal.

**Figure 3:**
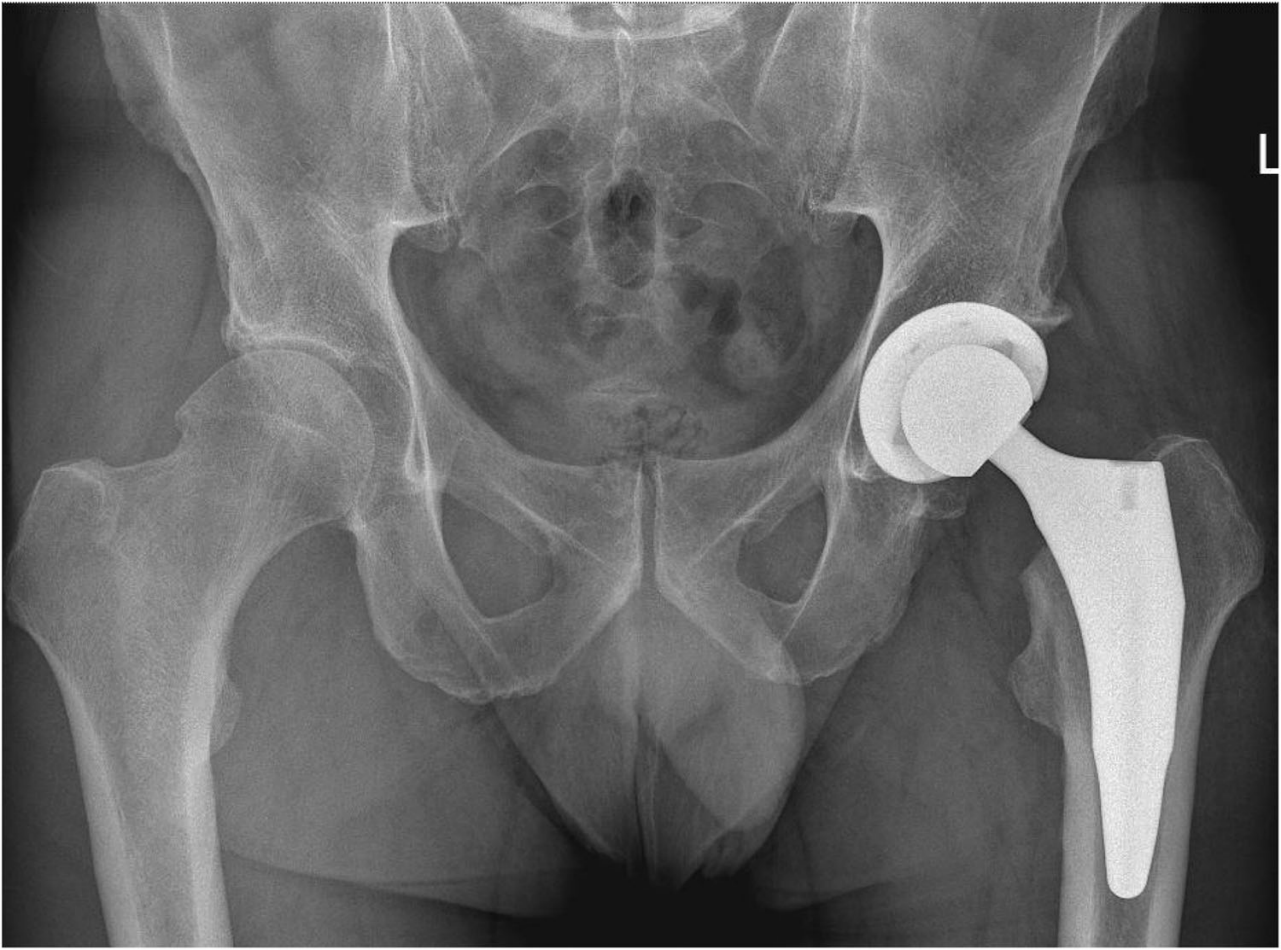
Two‐year postoperative radiographic follow‐up of a lateralizing 3C cementless short femoral stem (CCD angle: 127.5°). CCD, Caput–Collum–Diaphyseal.

Preoperative planning was performed using the OsiriX DICOM Viewer (Pixmeo): femoral offset and the distance from the lesser trochanter to the head centre were measured on the contralateral hip to guide restoration of hip biomechanics intraoperatively [26, 27]. In every case, a 3C Hip Prosthesis System cementless short stem (Link Italia S.p.A.) was implanted (Figure 1). This is a straight short stem with tapered lateral shoulder, tapered lateral distal stem and proximal coating to encourage proximal bone ingrowth. The implant features a straight profile and a broad proximal medio‐lateral width, ensuring rotational stability and preventing subsidence. This stem is made from forged Ti6Al4V alloy: the proximal stem is coated by an osteoconductive double coating made from pure titanium plasma spray and calcium phosphate (TiCaP) and the distal stem is smooth to discourage bone integration. The standard configuration features a Caput–Collum–Diaphyseal (CCD) angle of 131° (Figure 2), while the lateralized option decreases the CCD angle to 127.5° and increases femoral offset by 5 mm (Figure 3). Both versions maintain identical vertical height, with no change in the level of the neck cut. The standard and lateralized stems are available in 13 sizes each, whose dimensions and offset increase proportionately with increasing size. All procedures were carried out under spinal anaesthesia through a posterolateral approach. The height of the femoral neck osteotomy was preoperatively planned to ensure complete bony coverage of the double‐coated surface, optimizing both primary and secondary fixation. Then, after the neck osteotomy, the femur was prepared first by holding the knee flexed with the tibia in a vertical position. The starter device was introduced with the planned version for the definitive stem and the femoral rasps were inserted gradually, increasing the size until proper fit was achieved. The definitive anteversion of the stem was related to the native anatomy of the proximal femur. The selection between a standard or lateralized neck was based on the preoperative CCD angle measurements. The cup was positioned relative to the stem according to the femur‐first technique [26]. A cementless acetabular cup was used in all cases: a MobileLink cup (Waldemar Link GmbH & Co. KG) in 180 patients (90.0%) and an i1Cup (Link Italia S.p.A.) in 20 patients (10.0%). Based on the Dorr classification [38], femoral bone morphology was distributed as follows: Type A in 68 cases (34.0%), Type B in 126 cases (63.0%) and Type C in 6 cases (3.0%). The distribution of stem sizes was: size 2 in 1 case (0.5%), size 3 in 5 cases (2.5%), size 4 in 11 cases (5.5%), size 5 in 10 cases (5.0%), size 6 in 16 cases (8.0%), size 7 in 16 cases (8.0%), size 8 in 25 cases (12.5%), size 9 in 35 cases (17.5%), size 10 in 29 cases (14.5%), size 11 in 22 cases (11.0%), size 12 in 17 cases (8.5%) and size 13 in 13 cases (6.5%). Standard stem was used in 172 cases (86.0%), lateralizing stem in 28 cases (14.0%). The distribution of head sizes was: 28 mm (for dual mobility system) in 4 cases (2.0%), 32 mm in 131 cases (65.5%), 36 mm in 65 cases (32.5%). The distribution of head length was: S/− 4 mm in 42 cases (21.0%), M/+ 0 mm in 157 cases (78.5%); L/+ 4 mm in 1 case (0.5%). The bearing surface (ceramic‐on‐polyethylene or ceramic‐on‐ceramic) was selected in agreement with the patient after discussing the risks and benefits of each option in relation to their activity level. Distribution of bearing couples was: ceramic‐on‐ceramic in 10 cases (5.0%) and ceramic‐on‐polyethylene in 190 cases (95.0%). Range of motion exercises and partial weight‐bearing were initiated on postoperative Day 0, as soon as the effects of anaesthesia had resolved [9]. Partial weightbearing (50%) using two crutches was recommended for the first postoperative month.

### Clinical and radiographic evaluation

Patients were clinically assessed both preoperatively and postoperatively using the modified Harris Hip Score (mHHS), the Western Ontario and McMaster Universities Osteoarthritis Index (WOMAC) and the Visual Analogue Scale (VAS) for pain [1, 10, 12]. Preoperative patient‐reported outcome measures were prospectively collected during routine preoperative clinical evaluation for all patients undergoing THA at our institution and were retrospectively analysed for this study. Postoperative return to sports was evaluated using a custom, self‐reported questionnaire specifically designed for this study. Patients were asked whether they had resumed sporting activities compared with their pre‐disease level of participation, defined as the period before the onset of hip‐related symptoms that had led to activity limitation. The primary question was: ‘Have you returned to your pre‐disease level of sports participation?’. Possible responses were: (1) yes, full return; (2) partial return; (3) unable to return or (4) not applicable (no prior sports activity). All complications and reoperations were prospectively recorded.

At the final follow‐up, standardized radiographic assessment included an anteroposterior pelvic view centred midway between the anterior superior iliac spines and the pubic symphysis, and a true lateral view of the operated hip. Two fellowship‐trained orthopaedic surgeons (A.D. and M.R.) independently reviewed all radiographs. Discrepancies were resolved by consensus, and interobserver reliability was assessed using the intraclass correlation coefficient (ICC) and was 0.88. Radiographic evaluation focused on stem fixation, alignment and bone remodeling. Subsidence was measured as the change in vertical distance between the stem shoulder and the tip of the greater trochanter. Varus or valgus alignment was recorded relative to the femoral shaft axis: stem alignment was rated as valgus or varus if its deviation from the axis of the femoral shaft was more than 5°. Stress shielding and calcar resorption were graded according to Engh's classification [16], while cortical hypertrophy, spot welds and pedestal formation were noted when present. Radiolucent lines (RLLs) were assessed in the seven Gruen zones, and loosening was defined as progressive migration or a continuous radiolucency ≥2 mm. Heterotopic ossifications were classified following Brooker's classification [7].

### Data analysis

Implant survivorship was estimated using Kaplan–Meier methodology, with revision for any cause defined as the endpoint. Patients lost to follow‐up or deceased without a recorded date of death were censored at their last available evaluation. Continuous variables are presented as mean ± standard deviation or range (minimum–maximum), and categorical variables as absolute values and percentages. Crude event proportions were calculated for complications and revisions. To explore potential informative censoring, baseline characteristics were compared between patients retained at ≥2 years and those lost or deceased. Group comparisons for continuous variables employed Student's *t* test or the Mann–Whitney *U* test, as appropriate; categorical data were analysed with the *χ* ^2^ or Fisher's exact test. Normality of distributions was verified using the D'Agostino–Pearson test. Depending on data distribution, paired or non‐parametric tests were applied. All analyses were carried out with EasyMedStat software (version 3.40; EasyMedStat; www.easymedstat.com; accessed 27 October 2025) and figures were generated using GraphPad Prism (version 10.0.0; GraphPad Software).

## RESULTS

### Survival analysis, complication and revision rates

**Figure 4:**
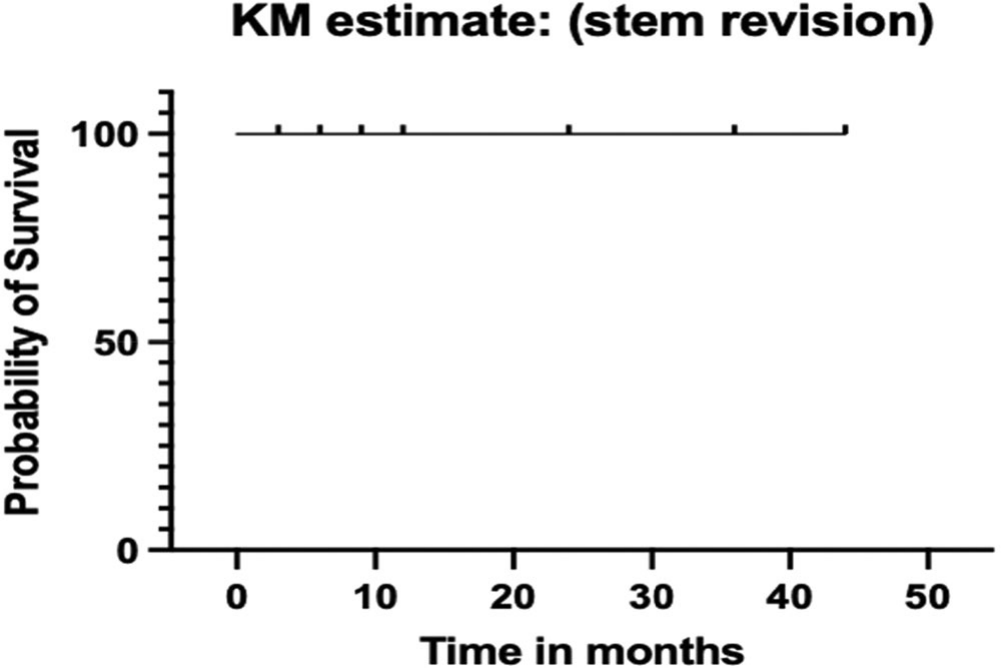
Kaplan–Meier (KM) survival curve with any revision of the implant as endpoint.

After a minimum follow‐up of 2 years, no patient died from causes related to the hip replacement, and only one patient (0.5%) required revision surgery consisting of a head exchange for instability. Ultimately, 200 patients were assessed clinically and radiographically at a mean follow‐up of 36.2 months (range: 24.0–44.0), yielding a follow‐up rate of 100.0%. Stem survivorship was 100.0%, as no femoral component required revision (Figure 4).

Three complications were recorded (complication rate 1.5%). In particular, one patient experienced an atraumatic posterior dislocation and underwent revision with a medium‐to‐long head exchange 23 days after the index surgery, one patient developed iliopsoas tendinopathy requiring arthroscopic iliopsoas tendon release and one patient reported a post‐traumatic ischio‐pubic ramus fracture, which was treated conservatively. Dislocation rate was 0.5%. Reoperation rate was 1.0%. In both cases, the stem was retained. No cases of intra‐ or postoperative femur fracture were reported. No cases of stem neck fracture were observed. None of the patients was revised for adverse local tissue reactions. No periprosthetic cup fractures were observed.

### Clinical outcomes

**Figure 5:**
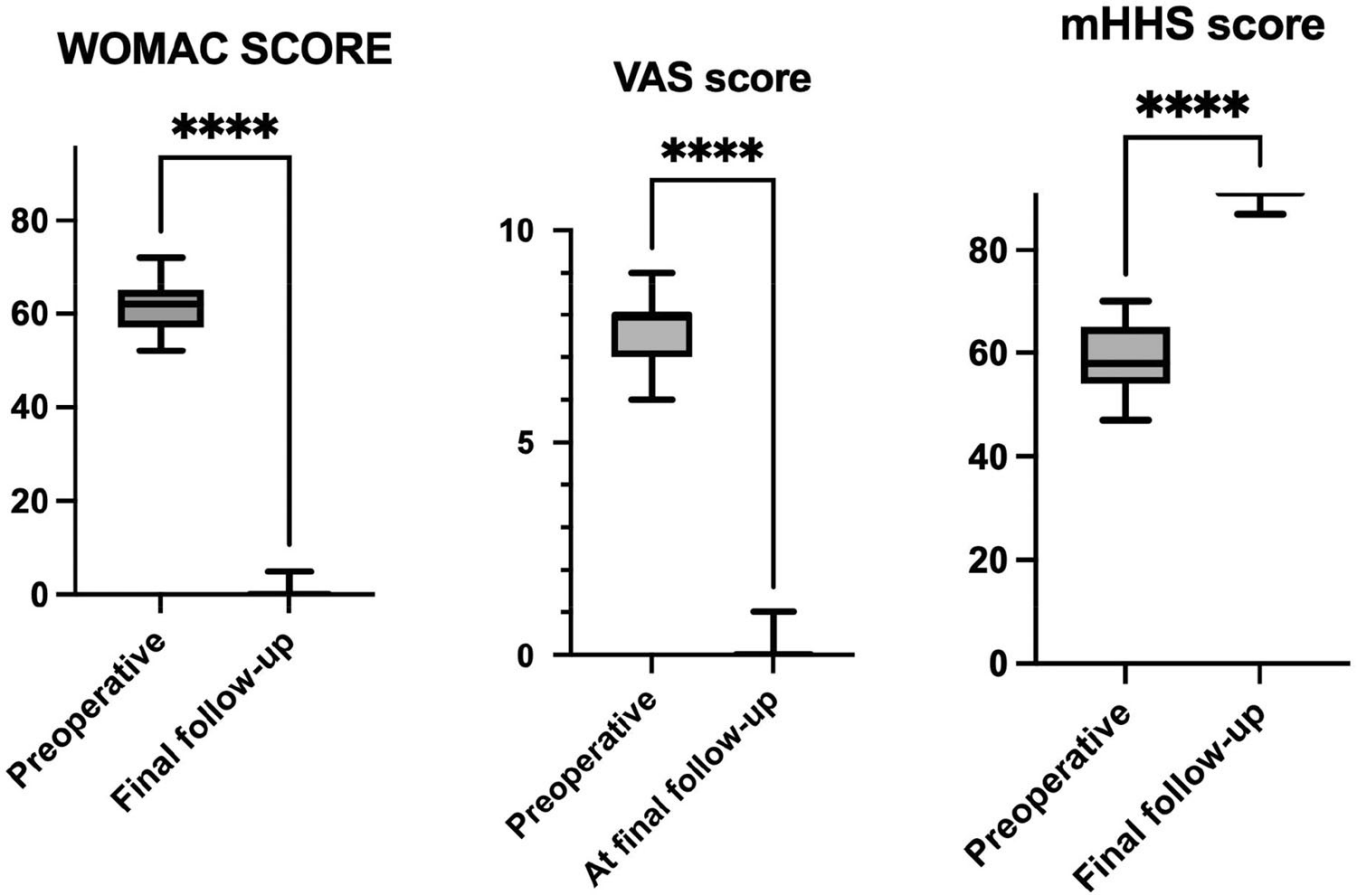
Box‐and‐whisker plots showing preoperative and final follow‐up patient‐reported outcome measures (PROMs), including WOMAC, modified Harris Hip Score (mHHS) and VAS pain score. Boxes represent the interquartile range with the median indicated; whiskers indicate the 10th and 90th percentiles. All scores improved significantly at final follow‐up (*p* < 0.0001). VAS, Visual Analogue Scale; WOMAC, Western Ontario and McMaster Universities Osteoarthritis Index.

All clinical scores improved significantly at the last follow‐up compared with the preoperative status, regardless of surgical indication. The mean preoperative WOMAC score was 60.5 ± 8.6 (range: 40.0–78.0), improving to 1.5 ± 3.3 postoperatively (range: 0.0–20.0) (*p* < 0.0001). The mean mHHS increased from 67.6 ± 9.1 (range: 31.0–81.0) preoperatively to 97.8 ± 3.1 (range: 79.0–100.0) at final follow‐up (*p* < 0.0001). Pain levels decreased substantially, with mean VAS scores improving from 7.6 ± 1.2 (range: 6.0–9.0) to 0.3 ± 0.7 (range: 0–4.0) (*p* < 0.0001). Regarding return to activity, overall return to sport was 85.0%: 95 patients (47.5%) achieved full return to their pre‐disease activity level, 75 patients (37.5%) reported a partial return, and 30 patients (15.0%) did not resume sports. Among the 200 clinically reviewed patients, trochanteric pain syndrome was reported in two cases (1.0%), and iliopsoas tendinitis occurred in two cases (1.0%); one was managed conservatively with anti‐inflammatory medication, while the other required arthroscopic iliopsoas tendon release. Thigh pain was not reported by any patient. Clinical outcomes are summarized in Table 2 and illustrated in Figure 5.

**Table 2:** Preoperative and postoperative clinical outcomes of the study population.

Clinical outcomes	*N*
Mean VAS (range)	
Pre	7.6 ± 1.2 (6.0–9.0)
Post	0.3 ± 0.7 (0.0–4.0), *p* < 0.0001
Mean WOMAC score (range)	
Pre	60.5 ± 8.6 (40.0–78.0)
Post	1.5 ± 3.3 (0.0–20.0), *p* < 0.0001
Mean mHHS (range)	
Pre	67.6 ± 9.1 (31.0–81.0)
Post	97.8 ± 3.1 (79.0–100.0), *p* < 0.0001
Return to sport (%)	170 (85.0%)
Full return	95 (47.5%)
Partial return	75 (37.5%)
Unable to return	30 (15.0%)

Abbreviations: mHHS, modified Harris Hip Score; VAS, Visual Analogue Scale; WOMAC, Western Ontario and McMaster Universities Osteoarthritis Index.

### Radiographic outcomes

**Figure 6:**
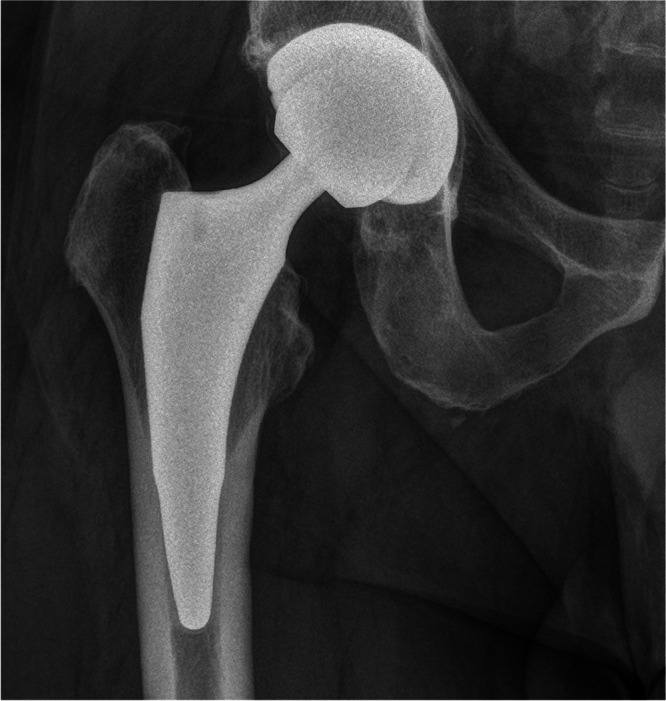
Two‐and‐a‐half‐year radiographic follow‐up showing stem radiolucent lines (RLLs) confined to Gruen Zone 4.

**Figure 7:**
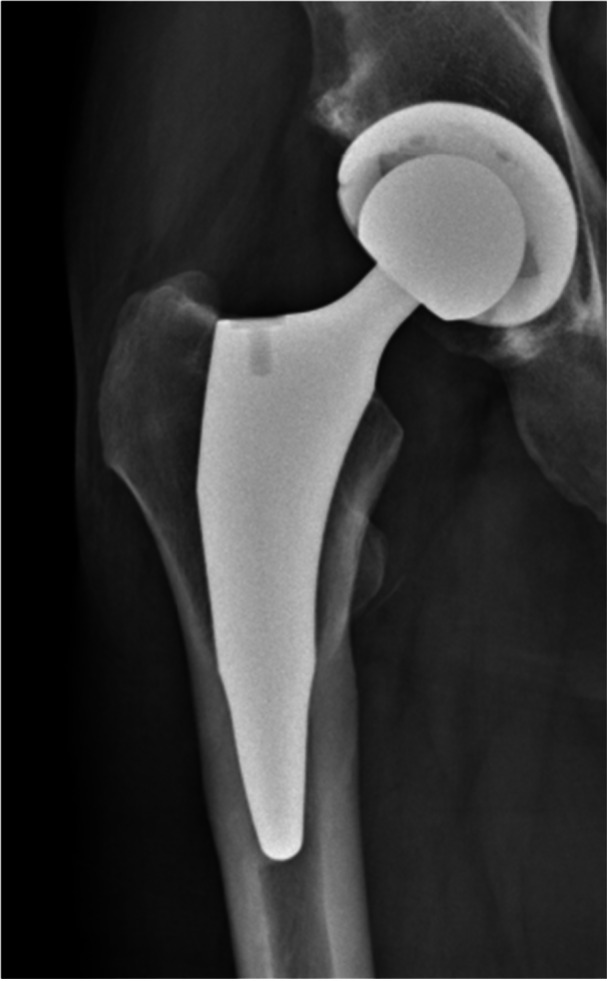
Two‐and‐a‐half‐year radiographic follow‐up showing cortical hypertrophy involving Gruen Zones 3–5.

At radiographic evaluation, assessment of stem alignment showed that 22 stems (11.0%) were implanted in varus and 10 stems (5.0%) in valgus relative to the diaphyseal axis, while the remaining stems were neutrally aligned. Stem RLLs were identified in 46 hips (23.0%). In most cases, RLLs were confined to Gruen Zone 4 (42 hips, 21.0%) and measured <2 mm (Figure 6). Two hips (1.0%) showed extension of a Zone 4 RLLs to the medial cortex (Zone 5), and the other two cases (1.0%) demonstrated thin (<2 mm) RLLs spanning Zones 3–5. Importantly, none of these findings were associated with signs of stem loosening. Cortical hypertrophy was observed in 8 hips (4.0%), consistently involving Gruen Zones 3–5 (Figure 7); these patients' femurs were classified as Dorr A in two cases and Dorr B in the other six cases. Stress shielding was classified as Grade I in 25 patients (12.5%) and Grade II in 10 patients (5.0%). No cases of severe stress shielding (Grades III and IV) were observed. Spot welds were present in 64 hips (32.0%). Heterotopic ossification occurred in two patients (1.0%) and were classified as Brooker Grade II. No cases of pedestal formation or stem subsidence were detected.

## DISCUSSION

To our knowledge, this is the first study to evaluate the clinical and radiographic outcomes of this specific short cementless stem in a consecutive series of primary THAs in young and active patients. At a minimum follow‐up of 2 years, the implant demonstrated excellent survivorship with no cases of aseptic stem loosening or clinically relevant subsidence. Clinical scores improved significantly, pain levels markedly decreased, and a substantial proportion of patients returned to their pre‐disease level of sports participation. Radiographically, the stem showed reliable metaphyseal fixation with low rates of cortical hypertrophy, limited and non‐progressive distal radiolucencies and no evidence of adverse bone remodeling. These results suggest that the combination of anatomical metaphyseal fit, selective proximal coating and favourable stem geometry can provide stable early fixation and high patient satisfaction in young and active individuals.

The stem demonstrated a survivorship of 100.0% at early‐term follow‐up, with no femoral component requiring revision. This result aligns with early‐ to mid‐term studies on short‐stem THA, which typically report survivorship rates between 96% and 99% at 3–5 years, confirming the reliability of modern metaphyseal‐anchored designs [17, 23, 25].

The clinical outcomes in this young and active cohort were excellent, with substantial improvements in all functional scores and pain metrics. Importantly, 85.0% of the patients in our series achieved a return to their pre‐disease level of activity, highlighting the capability of this implant to support the lifestyle demands of active individuals. Similarly, return to sport after short‐stem THA has been reported as highly favourable, with studies showing return‐to‐sport rates of 90%–98% and early resumption of activity in many patients. Ortmaier et al. [28] and Breuer et al. [6] demonstrated excellent recovery profiles, while Schmidutz et al. [31] confirmed that short stems allow patients to maintain active lifestyles, often with a shift toward lower‐impact sports. However, in our series patients were not stratified according to higher and lower‐impact sports.

Traditionally, mechanical complications such as stem subsidence, early loosening and intra‐ and postoperative femur fractures were described for short stems [4, 32, 37]. In our series, we did not observe intra‐ or postoperative femur fractures. However, these favourable results may apply only when short‐stem THA is used within the appropriate indications. Several authors have emphasized that short stems perform best in patients with good proximal bone stock, as metaphyseal anchorage requires adequate cancellous bone to achieve primary stability and reliable osseointegration [8, 38, 39]. Poor bone quality, particularly Dorr Type C femora, has been associated with higher risks of malalignment, subsidence and intraoperative fractures and is therefore considered a relative contraindication for many short‐stem designs [19, 29, 38]. In our cohort, 97.0% of patients were classified as Dorr A or B on preoperative radiographs, which likely contributed to the absence of intra‐ or postoperative femoral fractures and to the excellent stability observed at follow‐up. Moreover, no intra‐ or postoperative trochanteric fractures occurred, supporting the safety of this stem design. The tapered lateral shoulder of the stem is designed to preserve the greater trochanter by limiting excessive lateral cortical engagement, while still providing an adequate proximal mediolateral footprint for stable metaphyseal fixation. This helps reduce the risk of intraoperative trochanteric fractures, which have been reported with some short stems relying on broader lateral support [13, 25]. No cases of stem subsidence or early aseptic loosening were observed in our series. Stem subsidence beyond 6 months is considered abnormal and may indicate insufficient metaphyseal fixation [2, 5]. Early loosening is defined when progressive RLLs ≥2 mm, component migration or subsidence >2 mm occurred within the first 2 years postoperatively, indicating failure of primary fixation [14]. These findings suggest that the combination of anatomical metaphyseal fit and porous plasma‐spray coating provided stable fixation throughout follow‐up. The pronounced medial curvature offers effective metaphyseal support and load transfer, which is consistent with the limited calcar rounding and low incidence of cortical hypertrophy observed. Together, these design features promote both primary mechanical stability and reliable biological fixation by facilitating rapid bone ongrowth and osseointegration. Plasma‐sprayed titanium coatings have been shown to improve the quality and speed of bone–implant bonding, reduce micromotion at the interface and provide durable biological fixation over time [11, 30]. Indeed, spot welds were observed in around 30% of cases. Their presence aligns with reports on metaphyseal short stems, where spot welds commonly develop as bone adapts to proximal load transfer and ongrowth onto the coated surface [20, 36]. The frequency observed in our series falls within the range described in early‐ to mid‐term studies and reinforces the reliability of the metaphyseal anchorage achieved by this implant [20, 36]. RLLs at the distal stem tip were observed in 21.0% of our cases. These findings were thin, non‐progressive in appearance and not associated with stem migration or clinical symptoms. This is consistent with a tapered, uncoated distal stem that limits cortical contact, preserves metaphyseal load transfer and facilitates potential future revision. However, since we observed a 100.0% survivorship, we could not observe the potential benefits of this stem in revision THA. Similar radiographic patterns have been reported in studies of calcar‐guided and other metaphyseal‐anchoring short stems, where distal radiolucencies are considered a benign adaptive response in the unloaded diaphyseal region rather than a sign of aseptic loosening [24]. Kutzner et al. [24] described rare, non‐progressive RLLs ≤2 mm at the stem tip without negative impact on fixation or outcomes, consistent with the expected proximal load‐transfer behaviour of these designs. In our series, distal radiolucencies were not accompanied by pedestal formation or adverse proximal remodelling, further supporting their benign nature. However, because our study does not include serial radiographs over time, we cannot confirm their temporal evolution, and longer‐term, longitudinal imaging is required to determine whether these lines remain stable or progress.

Cortical hypertrophy in Gruen Zones 3–5 was detected in 4.0% of our cases. This finding is consistent with reports on modern metaphyseal‐engaging short stems, where distal cortical thickening is interpreted as an adaptive remodeling response to load distribution rather than an indication of pathological distal fixation. Prior studies on short stems have described similar low rates of focal hypertrophy (generally 4%–5%), typically without progression and with no impact on clinical outcomes or stem stability [22, 24]. In line with this evidence, the hypertrophy observed in our cohort was limited, non‐symptomatic and not associated with RLLs, pedestal formation or increased subsidence. These features suggest a benign remodeling phenomenon rather than a deviation from the intended proximal load‐transfer behaviour of the implant. Nevertheless, longer‐term follow‐up is required to confirm the stability of these findings over time.

In our series, no cases of acetabular loosening were observed, a finding that may be related to multiple factors, including the stem neck design and the consistent restoration of hip biomechanics achieved with a femur‐first technique, allowing accurate cup positioning and coplanarity [26]. Indeed, the flattened and tapered neck geometry may increase range of motion by reducing impingement, while the polished surface may help limit polyethylene abrasion and wear‐related risks.

This study has several limitations. First, its retrospective design is subject to selection and information bias, and causal inferences cannot be drawn. Second, all procedures were performed by a single high‐volume, fellowship‐trained surgeon, ensuring technical consistency but limiting the generalizability of our findings to different surgeons, centres and learning‐curve environments. Third, the cohort consisted predominantly of young and active patients, representing a favourable population for short stems; therefore, outcomes may not apply to older patients, those with poor bone quality or complex femoral morphologies. Fourth, we did not obtain serial radiographic series, which prevents us from assessing the temporal evolution of radiographic signs such as distal RLLs, cortical hypertrophy or long‐term remodeling patterns. Fifth, we did not include a comparison group using standard or longer stems, limiting our ability to contextualize the observed outcomes relative to alternative implants. Sixth, postoperative activity levels and return‐to‐sport data were based on self‐reported questionnaires. Return to sport was assessed using a custom, non‐validated questionnaire. Since activity levels were self‐reported and referred to the pre‐disease period, the results may be influenced by recall bias and subjective interpretation. Nevertheless, this approach was chosen to better reflect true functional recovery in a young and active population, as many patients had already reduced sports participation preoperatively due to symptoms. Future studies using validated activity scales and prospective data collection are needed to confirm these findings. Finally, the follow‐up remains mid‐term; longer‐term surveillance is needed to confirm the durability of osseointegration and to determine whether the favourable early fixation characteristics persist over time.

## AUTHOR CONTRIBUTIONS

*Conceptualization and design of the study*: Marco Minelli, Federico Della Rocca and Vincenzo Longobardi. *Methodology*: Giangregorio Madonna and Marco Minelli. *Validation*: Marco Minelli. *Formal analysis*: Marco Minelli and Mattia Andreotti. *Investigation*: Vincenzo Longobardi, Marco Minelli and Alessio D'Addona. *Data curation*: Marco Minelli, Marco Rosolani and Vincenzo Paolo Di Francia. *Writing—original draft preparation*: Marco Minelli. *Writing—review and editing*: Vincenzo Longobardi, Marco Minelli and Marco Rosolani. *Supervision*: Federico Della Rocca. All authors have read and agreed to the published version of the manuscript.

## CONFLICT OF INTEREST STATEMENT

Federico Della Rocca is a paid consultant for Link Italia S.p.A., Milan. The remaining authors declare no conflict of interest.

## ETHICS STATEMENT

This study was approved by the Institutional Board Committee (Protocol Number—618/17—IRCCS Istituto Clinico Humanitas).

## Data Availability

The authors have nothing to report.
